# Depression and the Risk of All-Cause and Cardiovascular Mortality in Patients With Chronic Obstructive Pulmonary Disease: A Study From NHANES 2005–2018

**DOI:** 10.1155/carj/8833533

**Published:** 2025-08-04

**Authors:** Hui Wei, Fachao Shi, Qin We, Bin Wang, Guoqin Qiu, Caoyang Fang

**Affiliations:** ^1^Department of Respiratory, Huzhou Central Hospital, Affiliated Central Hospital of Huzhou University, Affiliated Huzhou Hospital, Zhejiang University School of Medicine, Huzhou 313000, Zhejiang, China; ^2^Department of Cardiology, Maanshan People's Hospital, Maanshan 243000, Anhui, China; ^3^Department of Respiratory, Zhongda Hospital, Southeast University, Nanjing 210000, Jiangsu, China; ^4^Department of Emergency, First Affiliated Hospital of University of Science and Technology of China, Anhui Provincial Hospital, Hefei 230000, Anhui, China

**Keywords:** all-cause mortality, cardiovascular mortality, COPD, depression, NHANES, prognosis

## Abstract

**Objective:** At present, there is a lack of studies on depression and the likelihood for mortality among those suffering from chronic obstructive pulmonary disease (COPD). This research explores the connection between depression and the risks of overall mortality as well as cardiovascular mortality in individuals with COPD.

**Methods:** A total of 1336 COPD patients from seven cycles of the National Health and Nutrition Examination Survey (NHANES) conducted between 2005 and 2018 were selected. We created a multivariate Cox proportional hazards model and performed a subgroup analysis to investigate the connection between depression and both overall and cardiovascular mortality. Additionally, we used restricted cubic spline (RCS) curves to examine the relationship between depression and both overall and cardiovascular mortality to better reveal the association between the two. The Kaplan–Meier technique was employed to determine the likelihood of survival.

**Results:** Over the course of a mean follow-up period of 91 months, 1336 COPD patients were studied, of which 340 patients experienced overall mortality, and 82 had cardiovascular-related deaths. Using RCSs, we found that depression was positively correlated with both all-cause and cardiovascular mortality in COPD patients. In the multivariable-adjusted model, individuals suffering from moderate to severe depression had a greater likelihood of overall and cardiovascular mortality compared to those without depression. The results were consistent in subgroup analyses based on age, gender, body mass index (BMI), and poverty income ratio (PIR), and there was no significant interaction between these traits and depression (*p* for interaction > 0.05).

**Conclusion:** In COPD patients, depression is associated with higher risks of both cardiovascular and overall mortality. However, further validation of this finding is needed in large-scale prospective studies with sufficient follow-up time.

## 1. Introduction

Chronic obstructive pulmonary disease (COPD) is a common respiratory disease characterized by persistent airflow limitation [1]. In this study, the diagnosis of COPD was based on pulmonary function test results, i.e., FEV/FVC < 0.70, after bronchodilator treatment, which met the diagnostic criteria according to GOLD guidelines. Worldwide, COPD represents the fourth most common cause of death from chronic diseases, and there is evidence to suggest that by 2020, it will rise to become the fifth leading cause of global disease economic burden [2]. According to reports, having COPD raises the risk of issues like heart disease and stroke [3, 4]. Moreover, COPD patients have significantly increased risks of overall mortality and cardiovascular mortality. Therefore, identifying new risk factors early on is crucial for preventing, delaying, or reducing the progression of COPD and its related mortality.

Depression is a prevalent mental illness that impacts more than 15% of adult Americans [5]. According to the World Health Organization (WHO), depression ranks third among global disease burdens. Compared to the general population, those with COPD are more likely to exhibit signs of depression, and these symptoms seem to impact prognosis, as they lead to reduced physical activity, aggravated breathing difficulties, increased exacerbation frequency, and increased use of healthcare resources [6, 7]. Furthermore, depression and anxiety can interfere with other risk variables, including tobacco use, and overall, they may lower people's quality of life. The presence of anxiety and depression often indicates poor medication and pulmonary rehabilitation compliance, decreased physical function and quality of life, increased acute exacerbation and hospitalization frequency, prolonged hospitalization time, and significantly increased mortality rates [8–10]. Currently, there is an absence of analysis on the correlation between depression and both overall and cardiovascular mortality in COPD patients. Thus, the goal of this research is to ascertain how depression predicts overall and cardiovascular death risks in individuals with COPD.

## 2. Materials and Methods

### 2.1. National Health and Nutrition Examination Survey (NHANES) Study Population

NHANES is a nationwide cross-sectional investigation carried out through the National Center for Health Statistics (NCHS) every 2 years, aimed at exploring the risk factors and prevalence of common diseases among Americans. Healthcare practitioners with specialized knowledge gather data for NHANES, which includes demographic data, socioeconomic data, dietary data, and health-related data.

**Figure 1:**
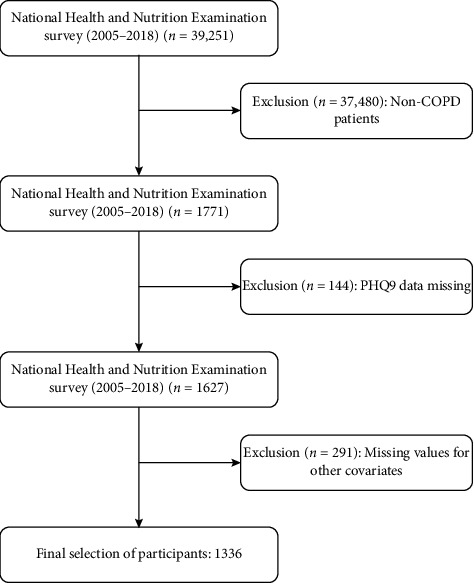
Study flowchart.

The following criteria were used to exclude individuals from this research: (1) age under 18 years old and (2) missing information on depression scores, pregnancy or gestational status, and death information. In addition, individuals lacking information on study variables such as education level, marital status, poverty income ratio (PIR), body mass index (BMI), and laboratory data were also excluded. Finally, 1336 eligible patients were selected. The research procedure was conducted in compliance with applicable legislation and recommendations. Before any data were collected, NHANES asked each participant to sign a written consent form to guarantee their consent. Considering the public nature of NHANES data and the openness and originality of research, the Ethics Committee of Huzhou Central Hospital exempted this study from the requirement for ethics approval. The specific research process can be found in Figure 1.

### 2.2. Definition of COPD and Assessment of Depression in NHANES

In this study, a validated depression screening tool, the PHQ-9, was used to assess the participants' depression status [11]. The PHQ-9 had four-point ratings for each symptom item, with scores spanning 0 (“*not at all*”) to 3 (“*nearly every day*”), and an overall score that varied between 0 and 27. Based on the total score categories from previous studies, participants were classified into no [0–4], mild [5–9], moderate [10–14], and severe [15–27] depression groups [12]. The total score was incorporated as a continuous parameter for RCS analysis within the study.

Diagnosis of COPD was based on the following criteria: Pulmonary function tests: FEV/FVC < 0.70 after bronchodilator treatment (mandatory, GOLD criteria fulfilled). Physician diagnosis: The patient was diagnosed with emphysema (mcq160g, mcq160p) by a physician or other healthcare provider. Additional conditions: age ≥ 40 years, and any of the following: (a) use of inhaled corticosteroids, mast cell stabilizers, leukotriene modifiers, or selective phosphodiesterase-4 inhibitors; (b) history of smoking; and (c) chronic bronchitis [13, 14].

### 2.3. NHANES Death-Related Information

Both cardiovascular and all-cause mortality were included in the investigation's findings. A probabilistic matching technique was utilized to ascertain the patients' mortality status after comparing the mortality data during National Death Index death certificates with the NHANES mortality data collected from 2005 to 2018. Cardiovascular mortality was estimated based on the codes in the International Classification of Diseases, 10th revision (ICD-10), which includes I00-I09, I11, I13, I20-I51, as well as I60-I69 codes. Follow-up ended either at the time of death or on December 31, 2019.

### 2.4. NHANES Covariates

Considering previous studies and clinical experience, we included covariates that might be associated with COPD mortality, including age, sex, race, education level, marital status, PIR, diabetes, hypertension, BMI, as well as smoking and alcohol consumption status. The race categories included non-Hispanic blacks and whites, Mexican Americans, and additional races. Three PIR ranges were identified: < 1.0, 1.0–3.0, and > 3.0. There were four categories for marital status: married, divorced, never married, and others. Three categories for the education level were established: below high school, high school or equivalent, and college or higher. In reference to smoking status, three categories were identified (1): Individuals who have never smoked a maximum of 100 cigarettes during their lifetime, individuals who have smoked over 100 cigarettes during their lifetime but are not currently smokers, and (3) individuals who have smoked higher than 100 cigarettes during their lifetime and are currently smoking either occasionally or daily. Alcohol consumption status was classified into never drank (less than 12 times in a lifetime), former drinker (did not drink in the past year but drank ≤ 12 times in the past year or drank ≤ 12 times in the past year but did not drink in the past year), heavy drinking (≥ 3 drinks a day for women and ≥ 4 for men per day, or binge drinking on five or more days a month), moderate drinking (≥ 2 drinks for women and ≥ 3 for men per day, as well as binge drinking on two or more days per month), and mild drinking (not fulfilling the prerequisites listed above). BMI was calculated as weight (kg)/height^2^ (m^2^), where a BMI of less than 25 kg/m^2^ was regarded as normal, a BMI of 25 kg/m^2^ to less than 30 kg/m^2^ as overweight, and a BMI of more than 30 kg/m^2^ as obese. Additionally, laboratory examinations included neutrophils, lymphocytes, hemoglobin, platelets, glycosylated hemoglobin (HbA1c), creatinine, uric acid, and blood urea nitrogen.

### 2.5. Statistical Analysis

The statistical analysis in the present study was carried out using R software (Version 4.3.2, https://www.r-project.org). We used the MEC sample weights (WTMEC2YR/7) to perform weighted analyses for all data. The means (SE) of continuous variables were displayed, while the frequencies (%) of categorical variables were reported. Prior to performing multivariate Cox regression analysis, we assessed collinearity between covariates using the variance inflation factor (VIF). VIF values greater than 5 are considered indicative of collinearity issues. If collinearity exists, we will remove variables with higher VIF values to avoid the effect of multicollinearity on the model results. We investigated the association between depression and the likelihood of cardiovascular and all-cause mortality using multivariable Cox proportional hazards regression models. Model 1 was unadjusted; Model 2 corrected for age, race, sex, marital status, education level, smoking, and alcohol consumption; and Model 3 corrected for age, sex, race, marital status, education level, smoking, alcohol consumption, PIR, and BMI. Additionally, we examined the association between depression scores and the likelihood of cardiovascular and all-cause mortality using RCS. In addition, we conducted stratified analyses for age, sex, BMI, and PIR. At the same time, a sensitivity analysis will be conducted on the imputed missing data using “mice.” In cases where the two-sided *p* value was below 0.05, the findings were deemed to be statistically significant.

## 3. Results

### 3.1. Initial Features of Depression Types in Individuals With COPD

The individuals' initial features are displayed in Table 1. A total of 340 individuals with COPD died from all causes among the 1336 individuals who had an average follow-up time of 91 months, with 82 of the deaths coming from cardiovascular causes. These 1336 people with COPD were 60.02 (0.44) years old on average. Significant variations were found in age, sex, BMI, marital status, education level, smoking, alcohol consumption, PIR, blood urea nitrogen, neutrophils, and lymphocyte levels (*p* < 0.05). Nevertheless, there were no substantial variations within race distribution, diabetes, hypertension, HbA1c, uric acid, creatinine, hemoglobin, and platelets (*p* > 0.05) among the patients.

### 3.2. Association of Depression Categories Alongside All-Cause and Cardiovascular Mortality Within Individuals Alongside COPD

#### 3.2.1. Association of Depression Categories Alongside All-Cause Mortality

**Figure 2:**
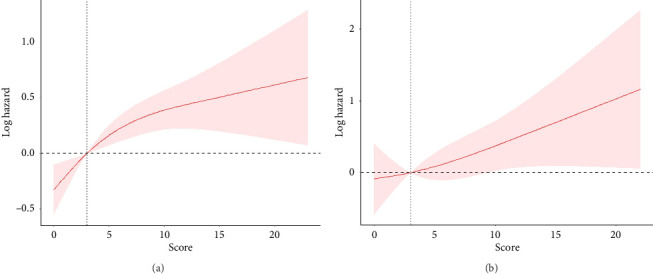
Association of depression with risk of all-cause mortality (a) and cardiovascular mortality (b) in COPD patients. Adjusted for age, sex, race, marital status, education level, smoking, alcohol consumption, PIR, and BMI.

**Figure 3:**
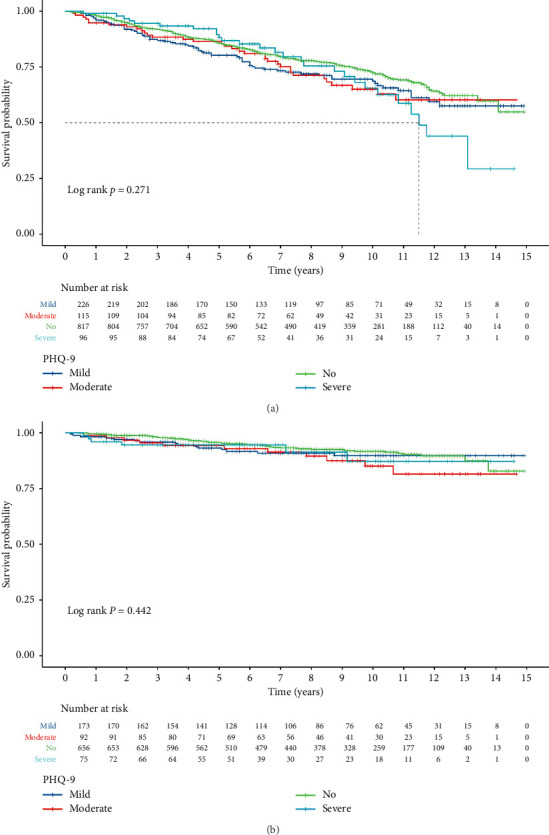
Kaplan–Meier survival curve analysis of depression and risk of (a) all-cause mortality and (b) cardiovascular mortality in patients with COPD.

During the follow-up of 1336 COPD patients, a total of 340 deaths from all causes were observed. In COPD patients, a positive association was shown using RCS analysis between the depression score and all-cause mortality (Figure 2(a)). In Model 2, we found a substantial decrease in the likelihood of all-cause mortality in people with COPD without depression (HR: 0.68, 95% CI: 0.49–0.94, *p*=0.002) (Table 2). In the multivariable-adjusted Model 3, COPD patients with moderate depression (HR: 3.71, 95% CI: 1.24–11.12; *p*=0.02) and severe depression (HR: 3.23, 95% CI: 0.73–14.35, *p*=0.12) revealed a notably elevated risk of death from all causes (Table 2). The Kaplan–Meier survival curve displayed a substantial drop in the survival rate in COPD patients with severe depression after 11.5 years (Figure 3(a)).

#### 3.2.2. Association of Depression Categories With Cardiovascular Death

RCS evaluation showed a positive correlation between the depression score and cardiovascular mortality in COPD patients (Figure 2(b)). Weighted multivariable Cox regression models showed that, in model 2, patients with mild depression who had COPD had a considerably greater chance of cardiovascular death (HR: 3.93, 95% CI: 1.26–12.24, *p*=0.02) (Table 2). In those suffering from COPD with moderate to severe depression, the likelihood of cardiovascular death was also elevated after multivariable correction (Table 2). The Kaplan–Meier survival curve displayed no substantial shift in the survival rate among COPD patients with different depression categories (Figure 3(b)).

### 3.3. Subgroup and Sensitivity Analyses

We also explored how different subgroups' risks of all-cause mortality related to depression categories. In male sufferers of COPD with severe depression, we observed a substantial rise in the likelihood of all-cause mortality following controlling for variables (HR: 1.352, 95% CI: 0.718–2.547, p for trend = 0.011) and PIR < 1 (HR: 2.259, 95% CI: 0.941–5.422, p for trend = 0.026). Moreover, there was a positive association found between the risk of all-cause death and depression categories (Table 3).

In the examination of the relationship between depression categories and the likelihood of cardiovascular mortality across various subgroups, we found a significant rise in the likelihood of cardiovascular mortality in female (HR: 14.401, 95% CI: 1.728–120.011, p for trend = 0.002) COPD patients with severe depression. Additionally, a nonlinear positive connection was found among the likelihood of cardiovascular mortality and depression categories (Table 3). In the subgroup analysis of all-cause and cardiovascular disease-related mortality, there was no interaction between each subgroup and depression categories (*p* > 0.05).

The sensitivity analysis showed that the relationship between depression categories and both all-cause mortality and cardiovascular mortality remained relatively stable (Table S1).

## 4. Discussion

To our knowledge, this is the only research that shows a nonlinear positive connection between depression and the likelihood of cardiovascular and all-cause mortality in individuals who have COPD. Our research shows a significant rise in the likelihood of cardiovascular and all-cause death in individuals with COPD who also have moderate to severe depression. Male and PIR < 1 COPD sufferers with severe depression had a definite increased chance of dying from all causes, while female COPD sufferers with severe depression had a definite increased chance of dying from cardiovascular causes, according to subgroup analysis.

The prevalence of COPD is rising worldwide. Between 1990 and 2010, the global prevalence of COPD among individuals aged 30 and over increased from 2.273 billion to 3.84 billion, and the global prevalence increased from 10.7% to 15.2% [15]. In China, the total number of COPD patients increased from 30.9 million to 51.52 million, a 66.73% increase [16]. As the population ages, COPD is becoming more common. In fact, among the elderly, its incidence rate might reach 16.9% [17]. By 2030, it is expected to overtake all other causes of death worldwide [18]. COPD has many comorbidities, and those with COPD have a markedly increased incidence of mental disorders, with depression having the highest incidence, followed by panic disorders, generalized anxiety disorders, and suicidal tendencies [19]. Research has indicated that depression prevalence varies between 6.7% and 58% in individuals with COPD [20]. A cross-sectional study in Pakistan found a depression prevalence rate of 57.2% [21], while in India, the depression prevalence rate was 22.7% [8], and in Shanghai, China, the depression prevalence rate among mild COPD patients was 13.4% [22]. Depression affects the quality of life and the overall survival rate [23, 24] and is linked to a higher chance of hospitalization for those with COPD as well as an aggravation of the disease [25].

Depression and COPD may interact, be separate conditions, or have a connection. Depression affects patients' self-management, increases the incidence and mortality of COPD, and exacerbates the risk of depression [26]. A systematic review analysis evaluated the relationship between COPD and depression, which included randomized controlled trials and used fixed-effect models for summary. According to the study, depression worsens the prognosis for COPD patients and steadily raises their risk of passing away. At the same time, COPD exacerbates the risk of depression [27]. Depression can increase the rate of readmission to hospital during acute exacerbations in COPD individuals and is a distinct risk indicator for short- and long-term readmissions. A study on the relationship between hospitalization rates in COPD patients and psychological disorders demonstrated that depression is correlated with hospitalization rates in COPD patients within 30 days [28]. Furthermore, there is a substantial correlation between the rate of hospitalization for acute exacerbations of COPD and medical expenditures. As a result of frequent readmissions, many hospitals are subject to restrictions on insurance and medical subsidies. Additionally, because depression is based on subjective feelings, it worsens respiratory symptoms, lowers quality of life and compliance in COPD patients, and influences the assessment of medical treatment, behavioral interventions, and patient benefits.

Research has shown that the prevalence of depression is higher among COPD patients who frequently experience exacerbations, and depression is more severe in patients during acute exacerbations of COPD [29]. Exacerbations of COPD appear to be associated with high mortality rates, with more than 26% of patients experiencing death within a year following an exacerbation that required hospitalization [30]. Many authors suggest that anxiety and depression are related to an increased risk of mortality after discharge [10, 31]. In our study, we found that COPD patients without depression had a significantly reduced risk of all-cause mortality (HR: 0.68, 95% CI: 0.49–0.94, *p*=0.002). After multivariate adjustment, COPD patients with moderate depression (HR: 3.71, 95% CI: 1.24–11.12; *p*=0.02) and those with severe depression (HR: 3.23, 95% CI: 0.73–14.35, *p*=0.12) showed a significantly increased risk of all-cause mortality. Additionally, the analysis of cardiovascular mortality risk in COPD patients revealed that those with moderate depression had a significantly increased risk of cardiovascular death (HR: 3.93, 95% CI: 1.26–12.24, *p*=0.02). Furthermore, after multivariate adjustment, both moderate and severe depression in COPD patients also corresponded to an increased risk of cardiovascular mortality. Subgroup analysis showed that the risk of cardiovascular mortality significantly increased in female patients with severe depression, while no interactions were observed in other subgroups. First, the changes in cardiovascular mortality may be related to the small sample size, particularly among female patients with severe depression, where insufficient sample size may lead to instability in statistical results. Second, the complexity of cardiovascular diseases and multifactorial influences may also result in the effects of depression on cardiovascular mortality being less pronounced than expected. We would like to emphasize that although there is an association between depression and cardiovascular disease, other factors (such as medication adherence, lifestyle, and comorbidities) may also play significant roles in this relationship. The Kaplan–Meier survival curve indicated a significant increase in all-cause mortality for COPD patients with severe depression after 11.5 years. However, the Kaplan–Meier survival curve showed no significant changes in cardiovascular mortality among COPD patients with different levels of depression. Due to the distinction between descriptive statistics (such as simple comparisons of survival rates) and inferential statistics (such as *p* values and confidence intervals), while descriptive statistics can provide preliminary observational results, inferential statistics are crucial for assessing whether group differences are significant. A *p* value of 0.271 indicates that there is not enough statistical evidence to suggest significant survival differences between the different categories of depression. This result suggests that the survival rates of patients with severe depression are statistically similar to those of patients with other categories, and therefore, we cannot reasonably claim a correlation between severe depression and lower mortality. Other factors that may affect survival rates, such as patients' comorbidities, treatment adherence, lifestyle, and socioeconomic factors, could differ among the various categories of depression, thereby influencing survival outcomes. The purpose of the Kaplan–Meier analysis is to compare survival distributions. Therefore, future research needs to consider these confounding factors in order to gain a more comprehensive understanding of the impact of depression on mortality.

Unlike the findings of our investigation, Zilz et al. discovered that depression and anxiety were not related to survival in patients with severe COPD [32]. There was no correlation between the death rates of patients with stable COPD and severe depression, according to the Maters et al. study [33]. The possible reason may be that our study population is different. Our study mainly focuses on nonhospitalized US populations, while the above studies are focused on hospitalized patients. Lower death rates may have resulted from the latter group receiving more specialized medical and care services following admission. Nevertheless, more investigation is still required to validate this finding.

The main strength of this study lies in its large overall sample size, sufficient follow-up time, reliable conclusions, and adequate statistical power. However, there are indeed some limitations to this research. First, although we made efforts to control for various potential confounding factors in our analysis, we cannot completely rule out the possibility that the PHQ-9 scores may be influenced by other unknown variables, such as medication adherence, levels of physical activity, and other mental health conditions (such as anxiety), which are indeed important factors. Therefore, future studies could further investigate the impact of these factors on the outcomes by collecting and analyzing data on medication adherence, physical activity levels, and other mental health conditions. Second, considering that a large number of participants lacking COPD and PHQ-9 data from NHANES were excluded from our observational analysis, this could lead to potential selection bias. Moreover, while our study findings provide important insights into the relationship between depression and mortality risk among COPD patients, caution must be exercised when applying these results to other populations, taking into account local healthcare practices and cultural contexts. We recommend that future studies be conducted in different cultural and healthcare settings to validate our findings and explore possible differences. Third, despite the good reliability and validity of the PHQ-9 in assessing depressive symptoms, its reliance on self-reported symptoms could lead to reporting bias. In future research, it would be beneficial to incorporate clinical assessments in addition to the self-reported PHQ-9 to provide a more comprehensive evaluation of depressive symptoms. Fourthly, PHQ-9 was assessed as cross-sectional data, and this study was an observational study design, so a causal relationship between depression and the risk of death in COPD patients could not be established. Lastly, during subgroup analyses, the sample sizes for certain categories were small, particularly for female patients with severe depression. This limitation may affect the stability and reliability of the analysis results; therefore, increasing the sample size in future studies would allow for a more comprehensive assessment of the impact of different categories of depression on the mortality risk in COPD patients.

## 5. Conclusions

The results of this study suggest that depressive mood is associated with an increased risk of all-cause mortality and cardiovascular mortality in COPD patients. Therefore, we suggest that clinicians should pay attention to the mental health of COPD patients and consider routine PHQ-9 screening and early recognition and intervention of depressive symptoms. In addition, existing integrated care models integrating respiratory and mental health, such as multidisciplinary collaborative management models, should be explored and promoted to improve the overall health and quality of life of COPD patients.

## Figures and Tables

**Table 1:** Baseline characteristics of CIOD patients by depression category.

Variables	Total	No	Mild	Moderate	Severe	*p* value
Age, years, mean (SE)	60.02 (0.44)	61.24 (0.53)	58.23 (0.97)	56.13 (1.23)	56.70 (1.54)	< 0.001
HbA1c, mean (SE)	5.88 (0.03)	5.84 (0.04)	5.89 (0.10)	5.89 (0.13)	6.23 (0.14)	0.07
Creatinine, μmol/L, mean (SE)	83.97 (0.96)	84.76 (1.18)	80.56 (1.63)	81.21 (2.69)	88.07 (3.69)	0.05
Uric acid, μmol/L, mean (SE)	333.85 (3.26)	336.23 (3.75)	329.38 (7.16)	313.48 (10.24)	343.50 (14.61)	0.22
BUN, mmol/L, mean (SE)	5.08 (0.07)	5.20 (0.08)	4.87 (0.18)	4.40 (0.25)	5.14 (0.24)	0.02
Neutrophils, × 10^9^/L, mean (SE)	4.73 (0.06)	4.60 (0.07)	4.76 (0.13)	5.04 (0.15)	5.65 (0.31)	0.01
Lymphocytes, × 10^9^/L, mean (SE)	2.10 (0.03)	1.99 (0.03)	2.36 (0.12)	2.35 (0.13)	2.30 (0.09)	< 0.001
Hemoglobin, g/dL, mean (SE)	14.33 (0.07)	14.41 (0.08)	14.14 (0.13)	14.18 (0.21)	14.16 (0.23)	0.16
Platelets, × 10^9^/L, mean (SE)	253.56 (2.47)	249.41 (3.01)	263.25 (5.97)	263.36 (10.74)	259.07 (7.70)	0.15
BMI, % (SE)						< 0.001
< 25	26.73 (0.02)	25.83 (2.03)	29.03 (4.25)	32.49 (6.30)	23.51 (5.31)	
25–30	33.45 (0.03)	38.63 (2.62)	24.43 (3.61)	23.38 (5.47)	15.96 (3.83)	
≥ 30	39.82 (0.03)	35.54 (2.30)	46.54 (3.91)	44.13 (6.00)	60.54 (5.57)	
Sex, % (SE)						< 0.001
Male	52.07 (0.03)	57.79 (2.33)	38.40 (4.01)	44.00 (6.13)	39.46 (5.97)	
Female	47.93 (0.03)	42.21 (2.33)	61.60 (4.01)	56.00 (6.13)	60.54 (5.97)	
Race, % (SE)						0.38
Mexican American	1.57 (0.00)	1.36 (0.32)	1.78 (0.54)	1.84 (0.81)	2.87 (1.24)	
Non-Hispanic black	6.41 (0.01)	5.78 (0.67)	7.4 (1.4)	8.07 (2.26)	8.32 (2.6)	
Non-Hispanic white	83.65 (0.05)	84.89 (1.54)	82.86 (2.47)	77.43 (3.84)	79.79 (3.78)	
Others	8.38 (0.01)	7.97 (1.25)	7.96 (1.85)	12.67 (2.87)	9.03 (2.54)	
Marital status, % (SE)						< 0.001
Married	58.04 (0.03)	63.54 (2.19)	50.11 (4.6)	40.36 (5.11)	42.47 (5.49)	
Never married	5.81 (0.01)	4.75 (0.8)	6.61 (1.81)	7.95 (2.04)	12.11 (3.8)	
Divorced	15.03 (0.01)	14.31 (1.63)	13.23 (2.69)	24.38 (4.54)	17.14 (4.15)	
Unmarried but have/had partner	21.12 (0.02)	17.4 (1.5)	30.05 (4.1)	27.3 (4.49)	28.27 (5.17)	
Education level, % (SE)						< 0.001
Less than high school	20.77 (0.02)	17.27 (1.64)	23.34 (3.46)	38.77 (4.98)	28.88 (5.83)	
High school or equivalent	25.39 (0.02)	23.69 (1.46)	31.52 (4.18)	27.29 (4.9)	24.11 (5.64)	
College or above	53.84 (0.03)	59.04 (2.11)	45.13 (4.46)	32.93 (4.81)	47.01 (6.34)	
Smoking, % (SE)						< 0.001
Never	17.36 (0.02)	19.15 (1.67)	14.47 (2.88)	9.51 (2.54)	15.33 (4.95)	
Former	46.16 (0.03)	52.41 (2.06)	38.14 (4.21)	21.98 (4.4)	30.2 (5.54)	
Now	36.49 (0.03)	28.44 (2.16)	47.39 (4.51)	68.51 (5.24)	54.47 (6.17)	
Alcohol consumption, % (SE)						0.01
Never	5.57 (0.01)	4.91 (0.77)	6.73 (1.86)	5.38 (2.11)	9.41 (3.98)	
Former	25.79 (0.02)	24.01 (1.92)	25.56 (3.13)	36.82 (5.65)	32.59 (5.86)	
Mild	36.06 (0.03)	39.2 (2.46)	27.48 (3.78)	29.05 (5.12)	34.8 (6.55)	
Moderate	15.6 (0.02)	16.81 (1.91)	17.23 (2.85)	8.33 (2.65)	6.75 (2.4)	
Heavy	16.98 (0.02)	15.07 (1.75)	23 (4.08)	20.43 (4.1)	16.45 (4.58)	
Diabetes, % (SE)						0.44
Yes	25.1 (0.01)	23.29 (1.81)	26.91 (4.29)	29.3 (5.08)	34.03 (5.81)	
No	63.96 (0.04)	64.92 (1.96)	64.5 (4.31)	59.85 (5.29)	57.2 (6)	
Borderline	10.94 (0.01)	11.79 (1.53)	8.59 (1.96)	10.85 (2.94)	8.77 (3.29)	
Hypertension, % (SE)						0.27
Yes	57.54 (0.03)	55.93 (2.2)	58.64 (4.53)	58.51 (6.47)	69.96 (5.58)	
No	42.46 (0.03)	44.07 (2.2)	41.36 (4.53)	41.49 (6.47)	30.04 (5.58)	
PIR, % (SE)						< 0.001
< 1	16.09 (0.02)	10.15 (1.35)	22.67 (4.05)	38.2 (4.93)	34.98 (5.86)	
1–3	38.3 (0.02)	36.89 (2.08)	40.25 (4.4)	40.4 (5.14)	45.07 (5.54)	
> 3	45.61 (0.03)	52.97 (2.45)	37.08 (5.07)	21.4 (6.07)	19.95 (5.41)	

*Note:* Date are presented as mean (SE) or *n* (%). HbA1c: glycosylated hemoglobin.

Abbreviations: BMI, body mass index; BUN, blood urea nitrogen; PIR, poverty income ratio.

**Table 2:** Relationship between depression and all-cause mortality and cardiovascular mortality in patients with COPD.

Variables	Model 1		Model 2		Model 3	
	HR (95% CI)	*p* value	HR (95% CI)	*p* value	HR (95% CI)	*p* value
All-cause mortality						
Mild	Ref		Ref		Ref	
No	0.77 (0.57, 1.04)	0.09	0.68 (0.49, 0.95)	0.02	1.05 (0.38, 2.92)	0.92
Moderate	1.17 (0.68, 2.02)	0.56	1.28 (0.78, 2.11)	0.33	3.71 (1.24, 11.12)	0.02
Severe	1.52 (0.86, 2.67)	0.15	1.71 (0.9, 3.26)	0.1	3.23 (0.73, 14.35)	0.12
*p* for trend	0.413		0.455		0.367	
Cardiovascular mortality						
Mild	Ref		Ref		Ref	
No	1.07 (0.49, 2.33)	0.86	0.96 (0.37, 2.49)	0.93	0.74 (0.53, 1.04)	0.08
Moderate	2.72 (1, 7.42)	0.05	3.93 (1.26, 12.24)	0.02	1.16 (0.68, 1.96)	0.58
Severe	2.29 (0.65, 8.05)	0.2	3.23 (0.79, 13.3)	0.1	1.66 (0.91, 3.02)	0.1
*p* for trend	0.148		0.101		0.129	

*Note:* Model 1: no adjustments made; Model 2: adjusted for age, sex, race, marital status, education level, smoking, alcohol consumption; Model 3: adjusted for age, sex, race, marital status, education level, smoking, alcohol consumption, PIR, BMI. Ref.: reference.

Abbreviations: CI, confidence interval; HR, hazard ratio.

**Table 3:** Stratified analysis of risk of all-cause and cardiovascular mortality in patients with depression and COPD.

Variables	Mild	No	Moderate	Severe	*p* for trend	*p* for interaction
All-cause mortality						
Age						0.568
< 60	Ref	0.733 (0.350, 1.534)	1.272 (0.427, 3.783)	1.894 (0.783, 4.586)	0.345	
≥ 60	Ref	0.756 (0.505, 1.131)	0.877 (0.474, 1.625)	1.396 (0.619, 3.146)	0.988	
Sex						0.337
Male	Ref	0.570 (0.363, 0.893)	0.671 (0.308, 1.461)	1.352 (0.718, 2.547)	0.011	
Female	Ref	0.933 (0.605, 1.438)	1.469 (0.789, 2.734)	1.792 (0.712, 4.509)	0.196	
BMI						0.975
< 25	Ref	0.89 (0.560, 1.414)	1.119 (0.622, 2.020)	1.795 (0.695, 4.631)	0.568	
25–30	Ref	0.702 (0.349, 1.409)	1.148 (0.335, 3.934)	1.470 (0.492, 4.393)	0.064	
> 30	Ref	0.718 (0.386, 1.336)	1.325 (0.547, 3.211)	1.378 (0.595, 3.193)	0.568	
PIR						0.846
< 1	Ref	0.962 (0.471, 1.966)	2.026 (0.872, 4.709)	2.259 (0.941, 5.422)	0.026	
1–3	Ref	0.619 (0.389, 0.986)	0.759 (0.393, 1.466)	0.945 (0.462, 1.933)	0.504	
> 3	Ref	1.114 (0.526, 2.356)	1.874 (0.237, 14.800)	3.372 (1.067, 10.655)	0.196	
Cardiovascular mortality						
Age						0.720
< 60	Ref	0.765 (0.054, 10.806)	4.089 (0.274, 60.933)	3.928 (0.412, 37.408)	0.078	
≥ 60	Ref	0.756 (0.271, 2.112)	2.051 (0.544, 7.725)	1.849 (0.374, 9.148)	0.969	
Sex						0.239
Male	Ref	0.583 (0.200, 1.703)	1.53 (0.439, 5.337)	0.817 (0.144, 4.637)	0.237	
Female	Ref	2.153 (0.354, 13.099)	10.939 (1.117, 107.15)	14.401 (1.728, 120.011)	0.002	
BMI						0.065
< 25	Ref	1.486 (0.231, 9.584)	7.59 (0.839, 68.638)	42.774 (5.67, 322.68)	0.602	
25–30	Ref	0.416 (0.103, 1.684)	0.823 (0.119, 5.706)	—	0.549	
> 30	Ref	0.594 (0.126, 2.804)	2.46 (0.386, 15.687)	1.178 (0.092, 15.029)	0.195	
PIR						0.173
< 1	Ref	0.518 (0.090,2.977)	0.863 (0.185, 4.023)	0.168 (0.011, 2.659)	0.938	
1–3	Ref	0.528 (0.145, 1.916)	1.016 (0.168, 6.145)	1.749 (0.182, 16.776)	0.704	
> 3	Ref	0.752 (0.127, 4.440)	7.854 (0.444, 138.827)	14.247 (1.795, 114.08)	0.623	

*Note:* Adjusted for age, sex, race, marital status, education level, smoking, alcohol consumption, PIR, and BMI.

Abbreviations: BMI, body mass index; PIR, poverty income ratio.

## Data Availability

The raw data supporting the conclusions of this article will be made available by the authors, without undue reservation.

## Supporting Information

Supporting Table 1. Sensitivity Analysis of Depressive Categories Associated with All-Cause Death and Cardiovascular Death.
