# Supplementary material for: Depression and the Risk of All-Cause and Cardiovascular Mortality in Patients With Chronic Obstructive Pulmonary Disease: A Study From NHANES 2005–2018
Source: Can Respir J. 2025 Aug 4;2025:8833533. doi: 10.1155/carj/8833533 (PMC12339155; doi:10.1155/carj/8833533)
Supplement: Supporting Information — Additional supporting information can be found online in the Supporting Information section. [file 8833533.f1.doc]

**Supplementary Table 1.Sensitivity Analysis of Depressive Categories Associated with All-Cause Death and Cardiovascular Death**

| **Variables** | **Model 1** | | **Model 2** | | **Model 3** | |
| --- | --- | --- | --- | --- | --- | --- |
|  | **HR(95%CI)** | **P-value** | **HR(95%CI)** | **P-value** | **HR(95%CI)** | **P-value** |
| ***All-cause mortality*** | | | | | | |
| Mild | Ref | | Ref | | Ref | |
| No | 0.77(0.57,1.04) | 0.09 | 0.71(0.51,0.97) | 0.03 | 0.76(0.56,1.04) | 0.09 |
| Moderate | 1.10(0.65,1.88) | 0.73 | 1.20(0.74,1.96) | 0.46 | 1.09(0.66,1.79) | 0.75 |
| Severe | 1.57(0.92,2.67) | 0.10 | 1.80(0.99,3.27) | 0.05 | 1.75(0.99,3.11) | 0.06 |
| P for trend | 0.763 | | 0.692 | | 0.942 | |
| ***Cardiovascular mortality*** | | | | | | |
| Mild | Ref | | Ref | | REf | |
| No | 0.76(0.39,1.48) | 0.43 | 0.72(0.33,1.54) | 0.39 | 0.82(0.36,5.79) | 0.65 |
| Moderate | 1.81(0.74,4.42) | 0.19 | 2.31(0.86,6.26) | 0.10 | 2.19(0.83,5.79) | 0.11 |
| Severe | 1.65(0.55,4.96) | 0.37 | 2.32(0.73,7.34) | 0.15 | 2.36(0.69,8.13) | 0.17 |
| P for trend | 0.621 | | 0.681 | | 0.943 | |

**HR: hazard ratio, CI: confidence interval, Ref: reference**

**Model 1: No adjustments made;**

**Model 2: Adjusted for age, sex,race, marital,education,smoke,alcohol;**

**Model 3:Adjusted for age, sex, racemarital,education,smoke,alcohol,PIR,BMI;**
